# Children and Parental Barriers to Active Commuting to School: A Comparison Study

**DOI:** 10.3390/ijerph18052504

**Published:** 2021-03-03

**Authors:** María Jesús Aranda-Balboa, Palma Chillón, Romina Gisele Saucedo-Araujo, Javier Molina-García, Francisco Javier Huertas-Delgado

**Affiliations:** 1 PROFITH “PROmoting FITness and Health through Physical Activity” Research Group, Sport and Health University Research Institute (iMUDS), Department of Physical Education and Sports, Faculty of Sport Sciences, University of Granada, 18011 Granada, Spain; pchillon@ugr.es (P.C.); rgs@ugr.es (R.G.S.-A.); 2 AFIPS Research Group, Department of Teaching of Musical, Visual and Corporal Expression, University of Valencia, 46010 Valencia, Spain; Javier.molina@uv.es; 3 “La Inmaculada” Teacher Training Centre, University of Granada, 18013 Granada, Spain; fjhuertas@ugr.es

**Keywords:** family, youth, perceptions, active transport

## Abstract

The main objectives of this study were: to compare the barriers to active commuting to and from school (ACS) between children and their parents separately for children and adolescents; and to analyze the association between ACS and the children’s and parents’ barriers. A total of 401 child–parent pairs, from Granada, Jaén, Toledo and Valencia, self-reported, separately, their mode of commuting to school and work, respectively, and the children’s barriers to ACS. *T*-tests and chi-square tests were used to analyze the differences by age for continuous and categorical variables, respectively. Binary logistic regressions were performed to study the association between ACS barriers of children and parents and ACS. Both children and adolescents perceived higher physical and motivational barriers and social support barriers towards ACS than their parents (all *p* < 0.05). Additionally, the parents perceived higher distance, traffic safety, convenience, built environment, crime-related safety and weather as barriers towards ACS, than their children (all *p* < 0.05). Moreover, a higher perception of barriers was related to lower ACS. The results of our study showed the necessity of attenuating the perceptions of children and their parents in order to increase ACS. This is relevant to develop interventions in the specific contexts of each barrier and involving both populations.

## 1. Introduction

Physical activity provides health benefits, such as the reduction of cardiovascular risks [1], psychological benefits, improved academic and cognitive achievement [2] and in cognitive functions in patients with cystic fibrosis [3] and mental [4] and social health benefits [5]. The World Health Organization (WHO) recommends at least 60 min of moderate-to-vigorous physical activity per day to achieve benefits [6]. In this sense, it is crucial to develop an active living style, which means, according to Sallis (2006), including four domains of physical activity: active recreation, exercise, active transport and household and occupational activities [7]. Therefore, active commuting to and from school (ACS), which is defined as walking or cycling to school, would lead to increased physical activity in youth populations [8]. In addition to the increase in physical activity, ACS improves physical fitness and well-being [9]. Despite all these benefits, in recent decades, ACS has decreased worldwide [10,11], and specifically in Spain, from 61% to 46% in adolescents [12]. Consequently, it is necessary to reverse this trend to contribute to a healthier society.

In addition, there are different factors that determine ACS, such as personal, social and environmental factors [13,14]. In relation to the personal factors, the preferential mode of commuting may influence the choice of the mode of commuting to school and specifically cycling to school [15]. In addition, other relevant factors reported as barriers to ACS for children are social support (e.g., “Have not many friends in neighborhood”) or environmental factors, such as traffic [16].

On the other hand, the parental barriers perceived about their children’s ACS play an important role. The perception of the distance between the house and school and the presence of dangerous intersections [16,17], traffic and crime-related safety [18], bullying and the possibility of abductions [19,20] are perceived as barriers associated with the ACS of their children. These barriers differ by children’s age; parental concerns over children’s safety are higher in younger children and should be reduced when they move to adolescence, so they have more freedom to commute between school and home independent of adult supervision [21]. A study in Seattle identified that the design of the neighborhoods and parental concerns were significantly associated with children’s ACS [22], so it will be important to know what parental perceptions are associated with ACS.

Consequently, there is evidence about the association of parent´s and children´s barriers with the children´s ACS. However, few studies compared the barriers of parents and children towards active commuting to/from school to have a more global overview and take these factors into account, which may be more relevant to implement future and effective interventions to promote ACS. A study in Washington D.C. with a sample of 193 children and their parents found that the parents’ confidence in their children’s walking or bicycling to school was much greater than the children’s confidence ratings [23]. Lastly, a study from Gent analyzed the differences in barrier perceptions and their relationship with independent mobility, and parents reported a more negative perception of traffic and crime-related safety than their adolescents. Moreover, the parents’ perception was more strongly associated with independent mobility than the adolescents’ perceptions [24].

Consequently, the aims of this study were: to compare the barriers to active commuting to and from school (ACS) between children and their parents, separately, for children and adolescents and to analyze the association between ACS and the children’s and parents’ barriers reported by a questionnaire, separately, for children and adolescents [25,26,27].

## 2. Materials and Methods

### 2.1. Study Design and Participants

This is a cross-sectional study with child and parental participation. The data were collected between March of 2018 and March of 2020 as part of the Pedalea y Anda al Cole/Cycle and Walk to School (PACO) Study. The PACO Study analyzes the determinants and correlates of ACS and examines the effects of a school-based cycling intervention on adolescents’ cycling to school, as well as on physical activity. In the current study, we focus on the correlates of ACS based on children’s and parents’ perceived barriers to ACS.

Participants were selected in two cohorts: (1) one public secondary school and one public primary school from the city of Alhendín (Granada) selected by convenience (2018); (2) ten public secondary schools from four cities, Granada, Jaén, Toledo and Valencia (2019 and 2020), which were randomly selected. The procedure in the 1st cohort started with contacting the schools of Alhendín and having meetings with the school board teams to inform them about the project. Then, after the school accepted the participation in the project, parents signed the informed consents to participate. The procedure in the 2nd cohort started by randomly selecting 10 secondary schools from the overall public secondary schools from four Spanish cities (i.e., 3 from Granada, 3 from Jaén, 1 from Toledo and 3 from Valencia). Once the school was selected, the research staff contacted the school board team to arrange a meeting and explain the project. After the school accepted the participation, parents signed the informed consents to participate.

A total of 600 child–parent pairs were invited to participate in this study and 401 children (girls’ mean age: 13.04 ± 1.89 years old; boys’ mean age: 13.02 ± 1.90 years old) and their parents (mothers’ mean age: 43.50 ± 5.39 years old; fathers’ mean age: 45.14 ± 4.72 years old) completed the questionnaires—only those child–parent pairs where both completed the questionnaire were included. The ethics committee of the University of Granada approved the study design, study protocols and informed consent procedure (Reference: 162/CEIH/2016).

### 2.2. Measurements

#### 2.2.1. Sociodemographic Characteristics

Parents self-reported their gender, age, educational level and socioeconomic status. The educational level was categorized as non-university (primary school, secondary school, baccalaureate, technical training) or university (university training). The socioeconomic status was categorized as low when the parents selected answers from none, <EUR 499, EUR 500–999, EUR 1000–1499 to EUR 1500–1999, or high when parents selected answers from EUR 2000–2499, EUR 2500–2999, EUR 3000–4999 to >EUR 5000.

#### 2.2.2. Mode of Commuting to/from School

The mode of commuting was extracted from the valid and reliable *“mode and frequency of commuting to and from school questionnaire”* [25,27], that was filled by children during their school schedule under supervision of the research team. The aim of the questionnaire is to determine the mode of commuting of children to/from school. The questions were: *“How do you usually get to school?”* and *“How do you usually get home from school?”*, and the possible answers were *walking, cycling, car, motorbike, scholar bus, public bus, metro/train or other*; only one option could be chosen. Children and adolescents were categorized as “active” if they reported walking or cycling as their mode of commuting and as “passive” if they answered car, motorbike, scholar bus, public bus, metro/train.

#### 2.2.3. Children´s Perceived Barriers (BATACE Questionnaire)

The children’s perceived barriers to ACS were assessed using the questionnaire “Barreras en el Transporte Activo al Centro Educativo” (BATACE), which has been validated in Spanish adolescents [28,29]. This questionnaire elicited information on barriers and perceptions of children go to/from school though a question (*“It’s hard for me to walk or bike to school because...:”)* and 18 items which refer to environmental safety (e.g., *there are one or more dangerous crossings*), autonomy (e.g., *I have too much stuff to carry*) or relatedness (e.g., *other children do not walk or bike*), among others. The participants had to rate how strongly they agreed with each statement through a Likert scale of 4 points (from “Strongly disagree” to “Strongly agree”).

This scale showed a good internal consistency for the subscale of environment/security barriers and for the planning/psychosocial barriers. In the same way, active commuting to school was related to the total scale, environment/security barriers and planning/psychosocial barriers [29].

#### 2.2.4. Parents’ Perceived Barriers (PABACS Questionnaire)

The parental perceived barriers to ACS were assessed using the Parental Perception of Barriers towards Active Commuting to School (PABACS), which has been validated in Spanish children and adolescents [26]. The question was formulated in this way: *“Here are some situations that might occur on a day-to-day basis. For each situation, please indicate how much you agree or disagree that it might affect your decision not to allow your child to walk/bike to or from school. (Please check only one option for each question.)”*. The scale includes 23 different items categorized as general barriers, including those common to both walking and cycling to school (e.g., *There is a long distance from home to school*), walking barriers, including those referring to walking (e.g., *there are no sidewalks or they are in poor condition*), and cycling barriers, including those referring to cycling (e.g., *there is no bike path or it is in poor condition*). The scale asked the participants to rate how strongly they agreed with each statement through a Likert scale of 4 points (from “Nothing” to “Substantially”).

This scale showed a good internal consistency for the overall question and for the three scales. The intra-class correlation values were moderate. The overall scale and the general and walking barrier scales showed a moderate to high validity to predict active modes of commuting [26].

#### 2.2.5. Comparison Procedure of Children’s and Parents’ Barriers

In order to be able to compare the children’s and parents’ barriers coming from the BATACE and PABACS questionnaires, respectively, the barriers to ACS were clustered into categories. These categories were proposed according to the scientific literature in a recent previous systematic review [30].

Following this categorization, we set 13 common barriers (i.e., categories) for both children and parents (see Table 1) to offer a common framework and be able to compare them. These 13 common barriers are grouped on the basis of general situations, walking situations and cycling situations.

### 2.3. Statistical Analysis

The descriptive data of the participants are presented as frequencies (and percentages) for categorical variables and mean and standard deviation for continuous variables. A Kolmogorov–Smirnov test was conducted to analyze the distribution and the results followed the normal distribution. Differences between age (child/adolescent) were calculated using a Student’s *t*-test for continuous variables and chi-square test for categorical variables. The internal consistency of barrier categories was checked by Cronbach’s alpha. To analyze the mean difference between children and their parents, and between adolescents and their parents, a *t*-test for independent samples was conducted. To establish the association between commuting to school and the barriers, several binary logistic regressions were performed. The mode of commuting was established as the dependent variable and each barrier was separately established as an independent variable. Consequently, a separate binary logistic regression was implemented for each barrier. All the analyses were performed with the statistical package SPSS for Windows version 23 (SPSS Inc., Chicago, IL, USA), establishing a level of statistical significance of *p* < 0.05.

## 3. Results

The descriptive data of participants and the differences between children and adolescents are presented in Table 2. The mean age of children was 13.26 ± 1.78 years old and the mean age of parents was 44.35 ± 5.54 years old. The children’s mode of commuting to/from school was mostly active (67.6%), and the parents’ mode of commuting to work was mainly passive (74.8%). Additionally, the educational level of parents was mainly non-university (62.6%) and the socioeconomic status was low (61.2%). The internal consistency showed Cronbach’s alpha values of 0.844 for general barriers, 0.489 for walking barriers and 0.524 for cycling barriers.

The Table 3 shows the parental barrier differences between children and parents by age group. The perception of barriers was different between children/adolescents and parents except for social support (walking) for both of them and physical and motivational barriers (cycling) in children (all *p* < 0.05). The parents reported higher importance for distance, traffic, convenience, built environment, crime-related safety and weather (all *p* < 0.05) than children and adolescents, whereas the children/adolescents reported higher importance of physical and motivational barriers and social support (all *p* < 0.05) than parents.

The results of Table 4 present the association between ACS and the barriers. In children, when they reported higher importance for the distance between home and school (OR = 0.411), traffic safety (OR = 0.492), convenience (OR = 0.518) and built environment (general) (OR = 0.661), the odds of actively commuting to school were lower. In relation to the parents of children, when they reported higher importance for distance (OR = 0.591), traffic safety (OR = 0.464) and convenience (OR = 0.389), the odds of actively commuting to school were lower. In adolescents, the odds of actively commuting to school were lower when they reported higher importance for distance (OR = 0.264), traffic safety (OR = 0.638), convenience (OR = 0.305), built environment (general) (OR = 0.666), crime-related safety (OR = 0.402), weather (OR = 0.737), built environment (walkability) (OR = 0.436) and built environment (bike) (OR = 0.641). In relation to the parents of adolescents, the odds of actively commuting to school were lower when the they reported higher importance for distance (OR = 0.476), traffic safety (OR = 0.635), convenience (OR = 0.327), built environment (general) (OR = 0.690), physical and motivational (general) (OR = 0.615) and physical and motivational (walk) (OR = 0.615).

## 4. Discussion

The main findings of this study were: [1] the most important barriers to ACS for children and adolescents were physical and motivational and social support barriers. By contrast, the most important barriers to their children’s ACS according to parents are the distance between home and school, traffic safety, convenience, built environment, crime-related safety and weather. [2] The ACS rates were lower when the children/adolescents and their parents reported higher perceived barriers to ACS.

The children and adolescents perceived more importance of the physical and motivational and social support barriers than their parents. The findings suggested that these groups of populations need physical or motivational encouragement to walk or cycle to school. Similarly, a study carried out in Australia found that different factors, such as social support, may influence children’s ACS [31]. Another study in the USA with adolescents reported that the absence of other children to walk with was related to ACS [28]. Consequently, the findings showed that it is more important for children and adolescents to have somebody to walk or cycle to school with than for the parents. Timperio et al. [31] highlight that having other children nearby may be important in all the strategies for increasing active commuting to school [31]. It is especially important when children grow because they become the decision maker of their mode of commuting. Finally, this higher importance for the children may be related to a greater importance of socialization for children and adolescents [32].

The parents showed greater concerns than children about the distance, traffic safety, convenience, built environment, crime-related safety and weather. In this sense, Greves et al. [20] found that parents and grandparents of children from 6 to 13 years old present crime-related safety, distance, built environment and weather as barriers to ACS of their children [20]. The same results were found in the studies of Carlson et al. [21] and Hume et al. [33], where the barriers of traffic safety and the built environment were perceived by parents of children and adolescents [17]. Parents would be more realistic about the barriers related to the natural and built environment as well as the traffic and the safety issues due to their greater experience because the ACS is a habit which is a representation of stimulus–response links and they are, in a sense, directly elicited by the environmental states or stimuli or contexts [34].

There are several child/adolescent and parental barriers associated with ACS. According to the results of our study, the barriers of distance, traffic safety, convenience and the built environment are associated with ACS, both for children and adolescents and their parents. Several studies confirm that the barriers, such as distance, traffic safety, convenience or the built environment are associated with ACS [30]. The barrier of distance is the main barrier associated with ACS [35] and is a predictor of the mode choice among adolescents [36]. Regarding the convenience barrier, a study carried out in Texas with 857 parents of children declared convenience as a barrier to active commuting to school of their children [37]. Additionally, the study of Timperio et al. [31] found the physical neighborhood environment to be a factor of influence on ACS, so, the improvement of urban design could be a strategy for increasing ACS. Finally, the traffic safety barrier is associated with ACS, because of the speed of traffic on the route to school; the amount of traffic; the safety at intersections; crossing problems; or the availability of crossing guards concern this population on the way to and from school [38].

In addition to the barriers mentioned in the previous paragraph, when the adolescents perceived crime-related safety and weather as barriers, ACS was lower. Crime-related safety is a barrier for adolescents, even more for boys than girls in their neighborhood [39]. Our study presented similar results to the study of Forman et al. [28], carried out in USA, where the barrier of crime-related safety was associated with ACS (e.g., “It is unsafe because of crime to walk or bike” or “I get bullied, teased, harassed along the way”) or the weather barrier (e.g., “It is not considered cool to walk or bike”). As the weather barrier could be affected by the location, in Spain, the main problem is the heat, as in the study of Herrador-Colmenero et al. [40], where the participants of this study perceived the weather as a barrier to commuting. Meanwhile, in other areas, such as the northern United States or Canada, extreme winter conditions (excess snow) are one of the main barriers associated with active commuting to school [22].

The results of our study showed the necessity of working with the perceptions of schoolchildren and parents in order to increase ACS. It is very important to develop interventions related to the specific contexts as barriers for parents of children and parents of adolescents are similar but not the same [17] and, consequently, interventions in school and high school may differ. In addition, strategies to improve the built environment infrastructure are necessary to encourage the behavior change. The perceptions of barriers of children and adolescents are susceptible to change as is the mode of commuting to school [41]. Consequently, the design of interventions and programs to promote ACS must be done with the objective of increasing the awareness in youth and their parents, and increasing the support for this behavior with regard to both parents’ and children’s similar perceived barriers.

So, reducing the perception of barriers will be necessary to design practical strategies, such as providing educational sessions where students learn what type of backpacks are best and least harmful for carrying weight or educational sessions about this behavior where students can learn the short- and long-term benefits of commuting actively. Additionally, another strategy could be the design and implementation of road safety education courses for students to learn how to get around safely by walking and cycling, so that parents may reduce their perception of barriers. In addition, increasing the rates of children who actively commute to school will be important to minimize the impact of noise exposure [42,43].

In our study, some limitations can be pointed out. Firstly, although in this study there is a great geographic diversity (i.e., four cities) within the same country, we cannot generalize the results because the sample was recruited only in Spain. It is necessary to highlight, as strengths of this study, that the sample of the study is a mixture of convenience and randomization samples and it was taken in two cohorts. Additionally, validated and reliable tools were used and a large population of child and parent pairs participated in this study. Finally, this is the first study that has compared the child/adolescent and parental barriers to our knowledge.

## 5. Conclusions

In conclusion, the children and adolescents perceived higher physical, motivational and social support barriers to ACS than their parents. The parents perceived a higher importance of distance, traffic safety, convenience, built environment, crime-related safety and weather than their children/adolescents. The distance between home and school, convenience, traffic safety, crime-related safety and built environment perceived by the children, adolescents and the parents were associated with a lower rate of ACS. In addition, when adolescents perceived crime-related safety and weather barriers, the rate of ACS was also lower. Therefore, it is necessary to design specific interventions that include both populations (parents and children) and focus on the most important barriers that they perceived towards ACS. To this end, it would be important that policy makers, administrations and schools provide the necessary support to implement such interventions.

## Figures and Tables

**Table 1:** Categorization of the barriers presented in the Barreras en el Transporte Activo al Centro Educativo (BATACE) and Parental Perception of Barriers towards Active Commuting to School (PABACS) scales.

BATACE	Categories	PABACS
General situations		
13. It is very far	Distance	1. There is a long distance from home to school
17. There are too much traffic	Traffic safety	2. There is a lot of traffic on the way to the school 4. The cars go very fast on the route to the school
9. It is easier to drive or to be taken 10. Too much advance planning is necessary	Convenience	12. It is more convenient to drive than to walk 18. It is more convenient to drive than to ride a bike
4. There are one or more dangerous crossings	Built environment	6. Lack of security at intersections and crossings
12. There are stray dogs 14. You would have to walk/cycle in places that would be unsafe due to crime or other crime related things (i.e., vandalism, graffiti, people drinking alcohol in public places) 3. The way does not have good lighting	Crime-related safety	7. There are no guards or police at crossings 8. There is violence and/or crime in the area
5. I get too hot and sweaty, or it always rains	Weather	9. It is very cold/hot 10.There is a lot of rain/snow
8. I have too much stuff to carry	Physical and motivational barrier	11. Your child carries a lot of weight in the backpack
**Walking barriers**		
1. There are no sidewalks or bike lanes 16. There are too many hills	Built environment (walk)	14. There are no sidewalks or they are in poor condition
6. Other children do not walk or bike	Social support (walk)	15. There are no other children to walk with 13. No other adults are walking the route from home to the school 17. No other parents are walking the children
2. The road is boring	Physical and motivational barriers (walk)	16. It is boring for your child to walk
**Cycling barriers**		
1. There are no sidewalks or bike lanes 18. Cycle lanes are occupied by people walking 11. There are no places to safely leave the bike	Built environment (bike)	20. There is no bike path or it is in poor condition 21. There is no place in the school to leave the bicycle
6. Other children do not walk or bike	Social support (bike)	19. There are no other adults who bike along the route from home to the school 22. There are no other children with whom to ride a bicycle 24. No other parents ride the children on a bicycle
2. The road is boring	Physical and motivational barriers (bike)	23. It is boring for your child to ride a bike

**Table 2:** Descriptive data of participants.

Sociodemographic Characteristics	All	Children	Adolescents	*p*
Children’s age	13.2 ± 1.7	10.7 ± 0.7	14.0 ± 1.1	**<0.001**
Commuting to/from school of children				
Active	265 (67.6)	59 (63.4)	206 (68.9)	0.326
Passive	127 (32.4)	34 (36.6)	93 (31.1)	
	**All**	**Parents of Children**	**Parents of Adolescents**	***p***
Parents’ age	44.3 ± 5.5	40.6 ± 5.1	45.4 ± 5.1	**<0.001**
Commuting to work				
Active	90 (25.2)	22 (25.9)	68 (25.0)	0.870
Passive	267 (74.8)	63 (74.1)	204 (75.0)	
Educational level				
Non-university	244 (62.6)	62 (67.4)	182 (61.1)	0.274
University	146 (37.4)	30 (32.6)	116 (38.9)	
Socioeconomic status				
Low (< EUR 1999)	219 (61.2)	51 (62.2)	168 (60.9)	0.829
High (> EUR 1999)	139 (38.8)	31 (37.8)	108 (39.1)	

Data in bold = Significant changes. *p*-value < 0.05.

**Table 3:** Comparison of children’s and parents’ barriers for children and adolescents.

Barrier Categories	Children	Parents of Children	Mean Difference	*p*
Distance general	1.83 ± 1.11	2.51 ± 1.04	0.67	**<0.001**
Traffic safety general	1.72 ± 1.04	2.76 ± 0.87	1.04	**<0.001**
Convenience general	1.76 ± 0.81	2.21 ± 1.05	0.45	**<0.001**
Built environment general	2.14 ± 1.26	2.69 ± 0.99	0.54	**<0.001**
Crime-related safety general	1.84 ± 0.87	2.12 ± 0.95	0.28	**0.037**
Weather general	1.46 ± 0.76	2.30 ± 0.76	0.84	**<0.001**
Physical and motivational barrier general	1.63 ± 1.03	1.33 ± 0.75	−0.30	**0.026**
Built environment (walk)	1.82 ± 0.90	2.33 ± 1.10	0.50	**0.001**
Social support (walk)	2.26 ± 1.25	2.15 ± 0.92	−0.10	0.507
Physical and motivational barriers (walk)	1.63 ± 1.03	1.33 ± 0.75	−0.30	**0.026**
Built environment (bike)	1.83 ± 0.77	2.34 ± 0.97	0.51	**<0.001**
Social support (bike)	2.26 ± 1.25	1.85 ± 1.04	−0.40	**0.019**
Physical and motivational barriers (bike)	1.63 ± 1.03	1.47 ± 0.85	−0.16	0.252
**Barrier Categories**	**Adolescents**	**Parents of Adolescents**	**Mean Difference**	***p***
Distance general	2.02 ± 1.26	2.62 ± 1.06	0.60	**<0.001**
Traffic safety general	1.89 ± 1.06	2.66 ± 0.94	0.76	**<0.001**
Convenience general	1.98 ± 0.91	2.16 ± 1.06	0.17	**0.030**
Built environment general	2.03 ± 1.10	2.67 ± 1.09	0.63	**<0.001**
Crime-related safety general	1.58 ± 0.64	2.12 ± 0.97	0.53	**<0.001**
Weather general	1.54 ± 0.82	2.10 ± 0.73	0.56	**<0.001**
Physical and motivational barrier general	1.85 ± 1.01	1.48 ± 0.77	−0.36	**<0.001**
Built environment (walk)	1.80 ± 0.89	2.30 ± 1.07	0.49	**<0.001**
Social support (walk)	2.11 ± 1.26	2.04 ± 0.77	−0.06	0.438
Physical and motivational barriers (walk)	1.85 ±1.01	1.48 ± 0.77	−0.36	**<0.001**
Built environment (bike)	1.82 ± 0.80	2.35 ± 0.95	0.53	**<0.001**
Social support (bike)	2.11 ± 1.26	1.72 ± 0.82	−0.39	**<0.001**
Physical and motivational barriers (bike)	1.85 ± 1.01	1.66 ± 0.91	−0.18	**0.018**

Data in bold = Significant changes. *p*-value < 0.05.

**Table 4:** Associations between active commuting to and from school (ACS) and the barriers, separated by children and adolescents.

Barrier Categories	Children			Parents of Children		
	Odd Ratio	CI 95%	*p*	Odds Ratio	CI 95%	*p*
Distance general	**0.411**	**0.265–0.638**	**<0.001**	**0.591**	**0.380–0.920**	**0.020**
Traffic safety general	**0.492**	**0.319–0.759**	**0.001**	**0.464**	**0.262–0.824**	**0.009**
Convenience general	**0.518**	**0.301–0.891**	**0.018**	**0.389**	**0.237–0.640**	**<0.001**
Built environment general	**0.661**	**0.471–0.928**	**0.017**	0.804	0.514–1.256	0.338
Crime-related safety general	1.043	0.640–1.698	0.867	1.056	0.660–1.689	0.820
Weather general	1.076	0.613–1.888	0.799	0.993	0.554–1.781	0.981
Physical and motivational barrier general	0.757	0.506–1.132	0.175	0.633	0.355–1.128	0.121
Built environment (walk)	0.794	0.499–1.263	0.329	0.910	0.610–1.359	0.646
Social support (walk)	1.284	0.907–1.816	0.158	0.781	0.485–1.258	0.309
Physical and motivational barriers (walk)	0.757	0.506–1.132	0.175	0.633	0.355–1.128	0.121
Built environment (bike)	0.672	0.390–1.156	0.151	1.074	0.682–1.691	0.758
Social support (bike)	1.284	0.907–1.816	0.158	0.796	0.525–1.209	0.284
Physical and motivational barriers (bike)	0.757	0.506–1.132	0.175	1.045	0.622–1.756	0.869
**Barrier Categories**	**Adolescents**			**Parents of Adolescents**		
	**Odds Ratio**	**CI 95%**	***p***	**Odds Ratio**	**CI 95%**	***p***
Distance general	**0.264**	**0.202–0.346**	**<0.001**	**0.476**	**0.364–0.623**	**<0.001**
Traffic safety general	**0.638**	**0.507–0.802**	**<0.001**	**0.635**	**0.481–0.838**	**0.001**
Convenience general	**0.305**	**0.220–0.422**	**<0.001**	**0.327**	**0.244–0.438**	**<0.001**
Built environment general	**0.666**	**0.533–0.831**	**<0.001**	**0.690**	**0.544–0.876**	**0.002**
Crime-related safety general	**0.402**	**0.270–0.598**	**<0.001**	1.087	0.841–1.406	0.522
Weather general	**0.737**	**0.554–0.981**	**0.037**	0.881	0.629–1.232	0.458
Physical and motivational barrier general	0.801	0.631–1.018	0.069	**0.615**	**0.451–0.838**	**0.002**
Built environment (walkability)	**0.436**	**0.488–0.587**	**<0.001**	0.951	0.755–1.198	0.670
Social support (walk)	1.069	0.876–1.303	0.512	1.022	0.740–1.410	0.896
Physical and motivational barriers (walk)	0.801	0.631–1.018	0.069	**0.615**	**0.451–0.838**	**0.002**
Built environment (bike)	**0.641**	**0.473–0.868**	**0.004**	1.198	0.913–1.572	0.191
Social support (bike)	1.069	0.876–1.303	0.512	0.828	0.612–1.121	0.223
Physical and motivational barriers (bike)	0.801	0.631–1.018	0.069	0.987	0.747–1.303	0.926

Data in bold = Significant changes. *p*-value < 0.05.

## Data Availability

The data will be available on request.
